# Supplementary figures and images for: Research and experimental verification on the mechanisms of cellular senescence in triple-negative breast cancer
Source: PeerJ. 2024 Feb 29;12:e16935. doi: 10.7717/peerj.16935 (PMC10909353; doi:10.7717/peerj.16935)

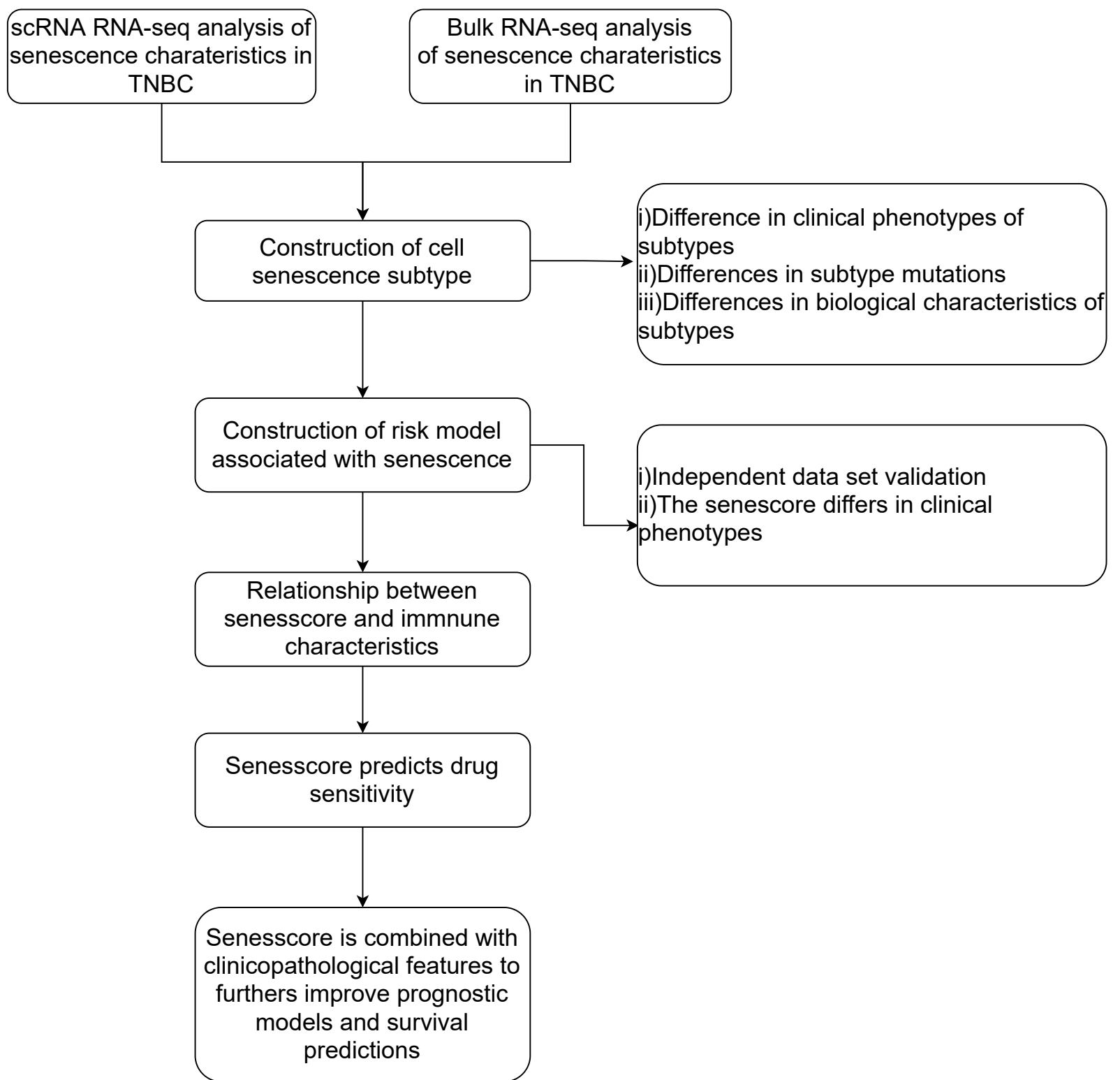

Supplement: Figure S1 [file peerj-12-16935-s001.pdf]

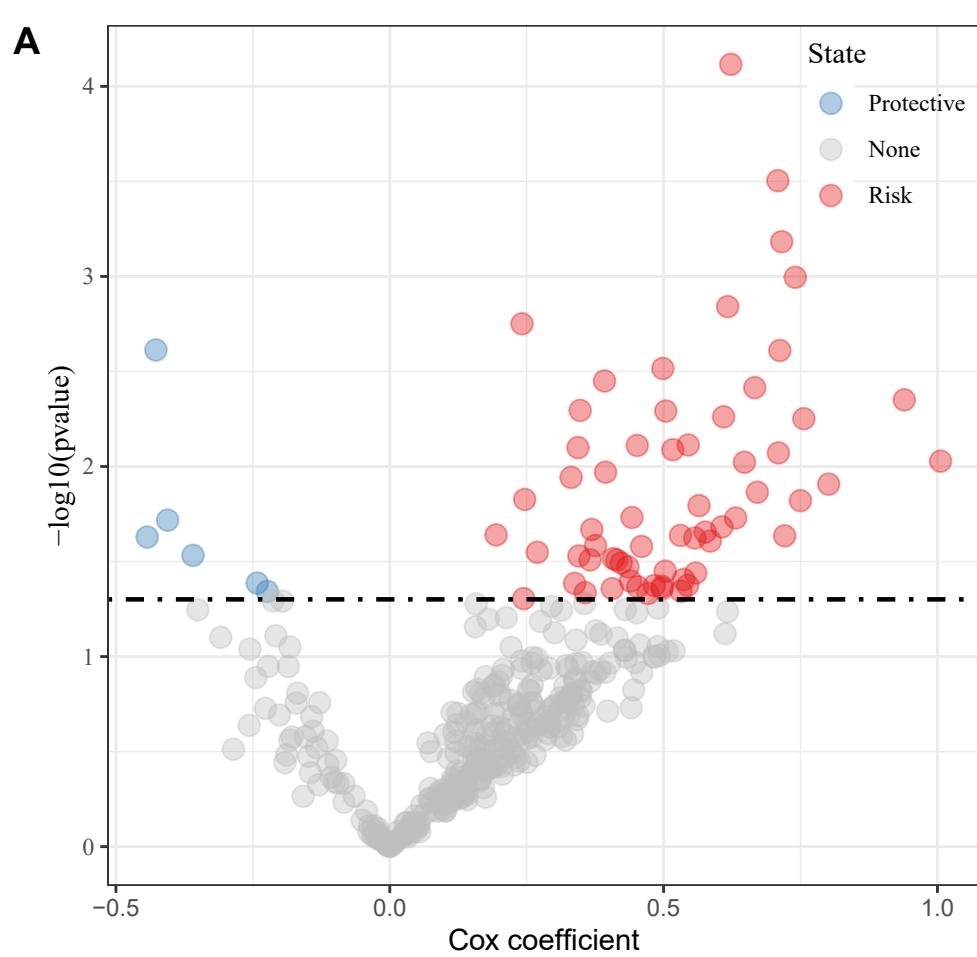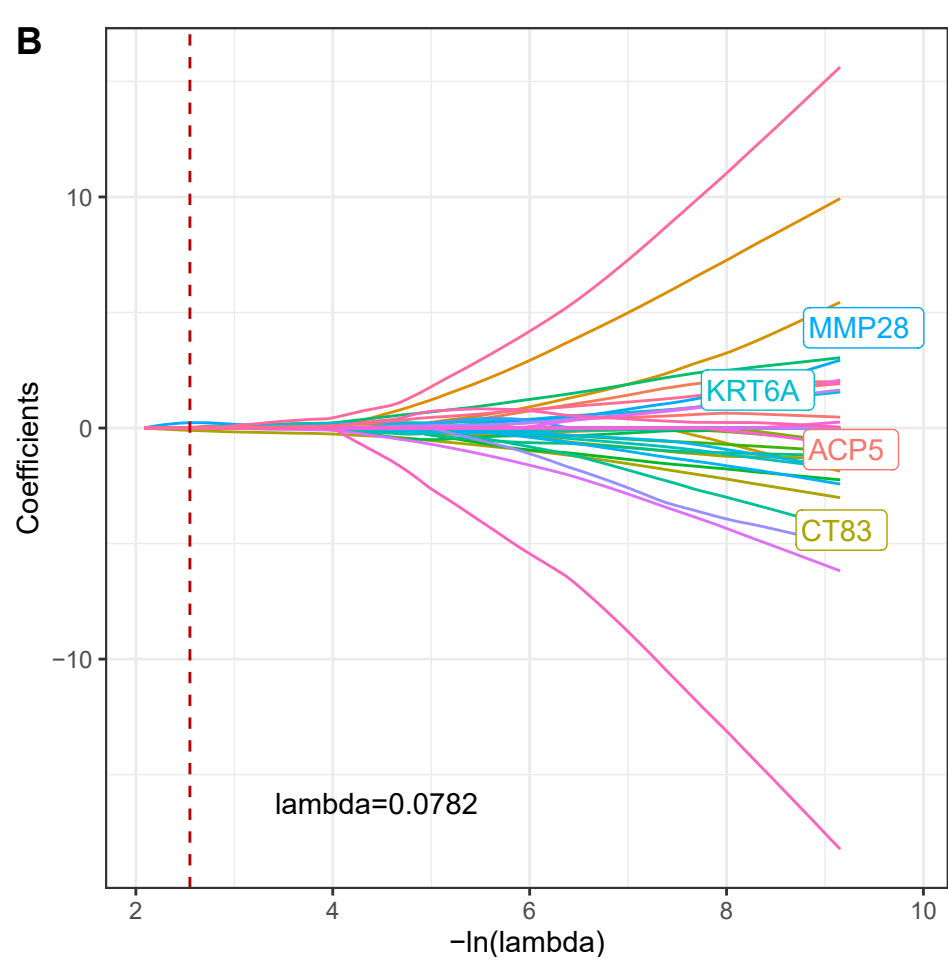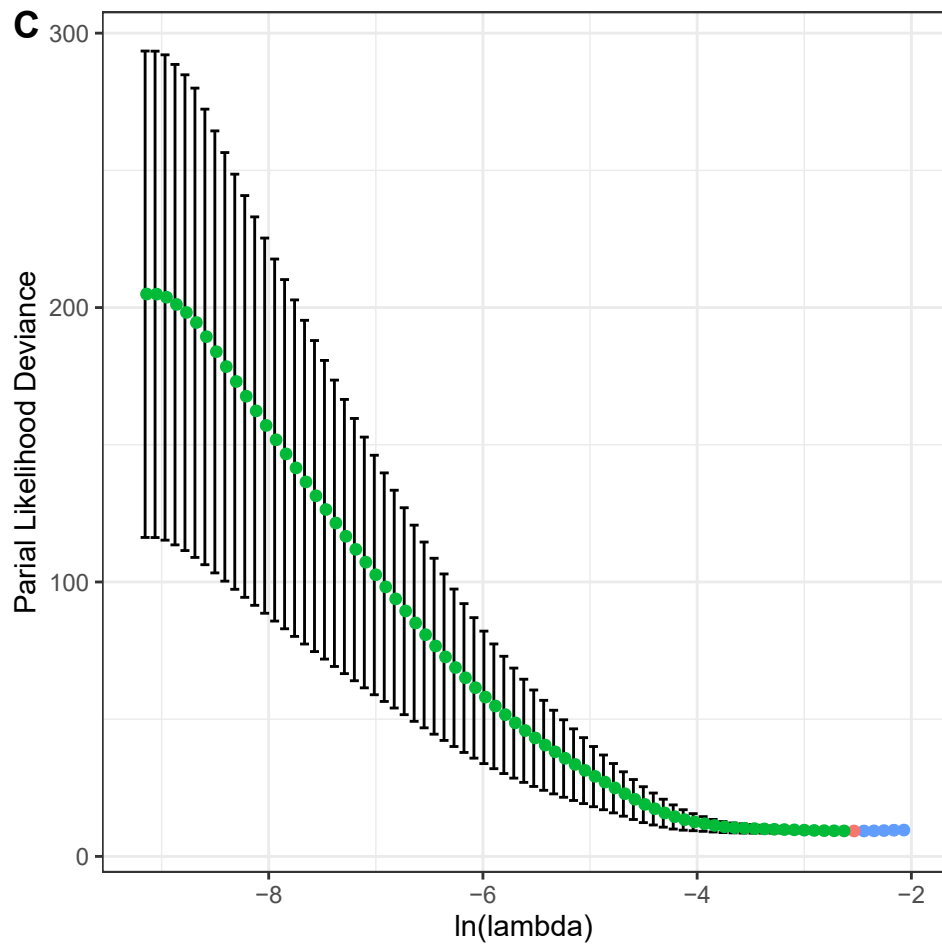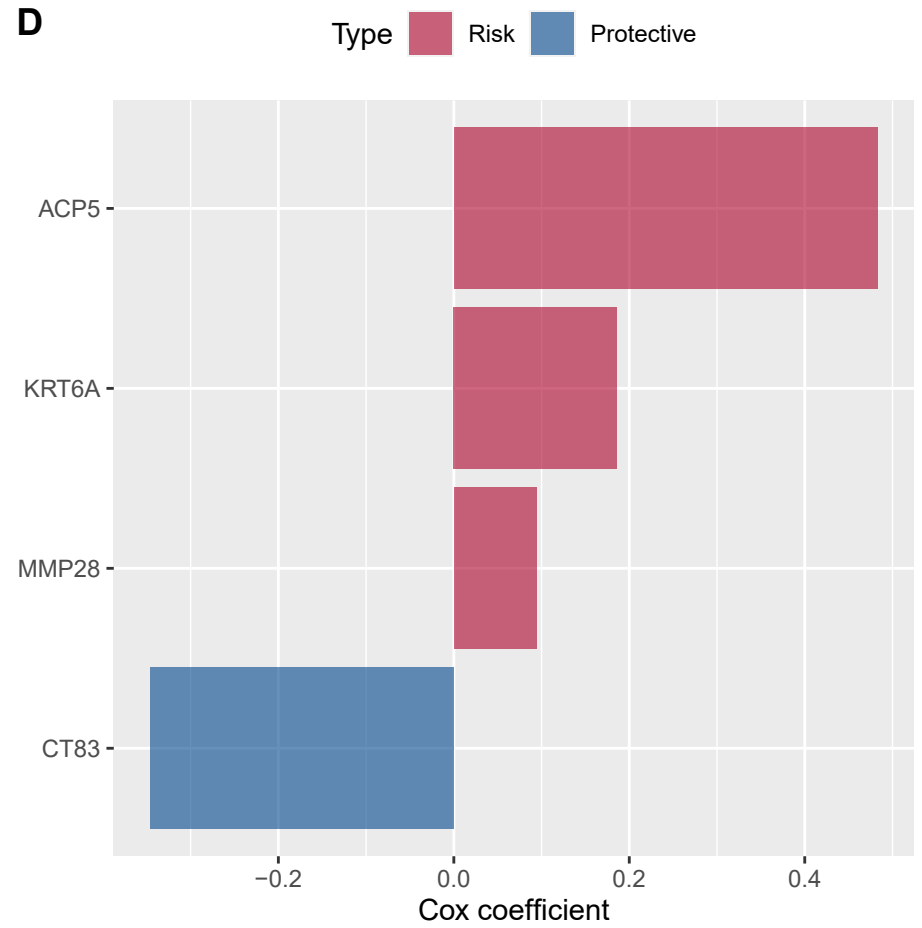

Supplement: Figure S3 — A: A total of 961 promising candidates were identified among the DEGs; B: Changing trajectory of each independent variable with lambda; C: Confidence interval for lambda; D: Coefficients of prognosis-related genes in multivariate Cox analysis. [file peerj-12-16935-s003.pdf]

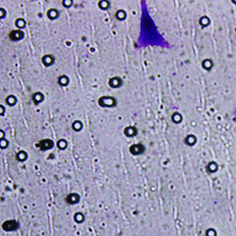

Supplement: Data S1 [file peerj-12-16935-s006.zip › The raw data of experiments/TRANSWELL/MDA-MB-231/invasion/si ACP5 1.jpg]

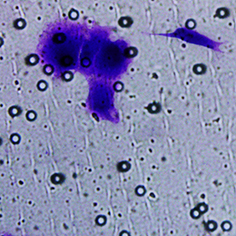

Supplement: Data S1 [file peerj-12-16935-s006.zip › The raw data of experiments/TRANSWELL/MDA-MB-231/invasion/si ACP5 2.jpg]

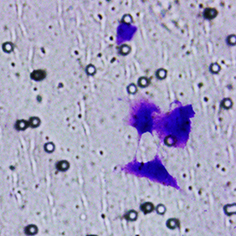

Supplement: Data S1 [file peerj-12-16935-s006.zip › The raw data of experiments/TRANSWELL/MDA-MB-231/invasion/si ACP5 3.jpg]

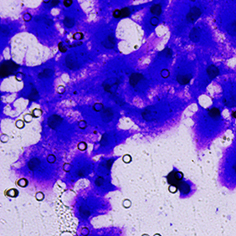

Supplement: Data S1 [file peerj-12-16935-s006.zip › The raw data of experiments/TRANSWELL/MDA-MB-231/invasion/si NC 1.jpg]

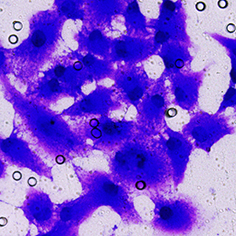

Supplement: Data S1 [file peerj-12-16935-s006.zip › The raw data of experiments/TRANSWELL/MDA-MB-231/invasion/si NC 2.jpg]

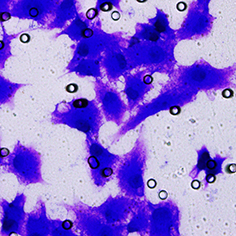

Supplement: Data S1 [file peerj-12-16935-s006.zip › The raw data of experiments/TRANSWELL/MDA-MB-231/invasion/si NC 3.jpg]

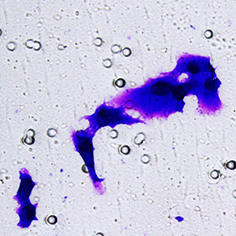

Supplement: Data S1 [file peerj-12-16935-s006.zip › The raw data of experiments/TRANSWELL/MDA-MB-231/migration/si ACP5 1.jpg]

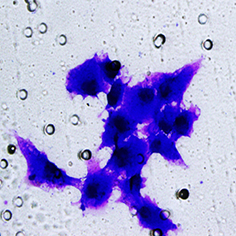

Supplement: Data S1 [file peerj-12-16935-s006.zip › The raw data of experiments/TRANSWELL/MDA-MB-231/migration/si ACP5 2.jpg]

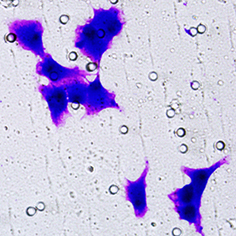

Supplement: Data S1 [file peerj-12-16935-s006.zip › The raw data of experiments/TRANSWELL/MDA-MB-231/migration/si ACP5 3.jpg]

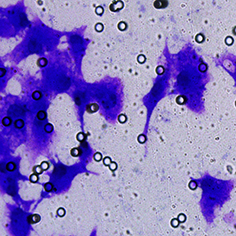

Supplement: Data S1 [file peerj-12-16935-s006.zip › The raw data of experiments/TRANSWELL/MDA-MB-231/migration/si NC 1.jpg]

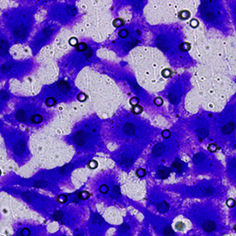

Supplement: Data S1 [file peerj-12-16935-s006.zip › The raw data of experiments/TRANSWELL/MDA-MB-231/migration/si NC 2.jpg]

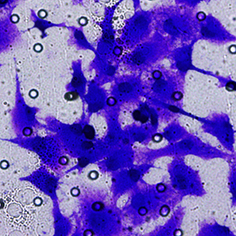

Supplement: Data S1 [file peerj-12-16935-s006.zip › The raw data of experiments/TRANSWELL/MDA-MB-231/migration/si NC 3.jpg]

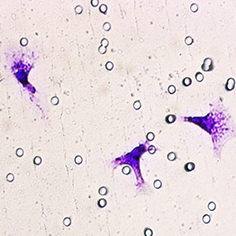

Supplement: Data S1 [file peerj-12-16935-s006.zip › The raw data of experiments/TRANSWELL/MDA-MB-468/invasion/si ACP5 1.jpg]

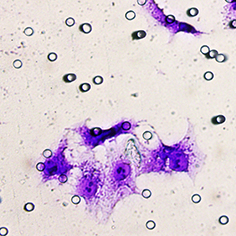

Supplement: Data S1 [file peerj-12-16935-s006.zip › The raw data of experiments/TRANSWELL/MDA-MB-468/invasion/si ACP5 2.jpg]

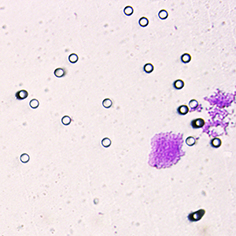

Supplement: Data S1 [file peerj-12-16935-s006.zip › The raw data of experiments/TRANSWELL/MDA-MB-468/invasion/si ACP5 3.jpg]

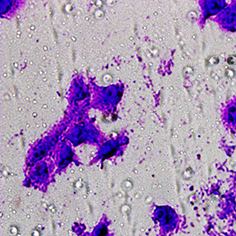

Supplement: Data S1 [file peerj-12-16935-s006.zip › The raw data of experiments/TRANSWELL/MDA-MB-468/invasion/si NC 1.jpg]

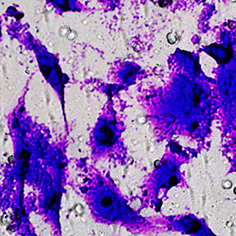

Supplement: Data S1 [file peerj-12-16935-s006.zip › The raw data of experiments/TRANSWELL/MDA-MB-468/invasion/si NC 2.jpg]

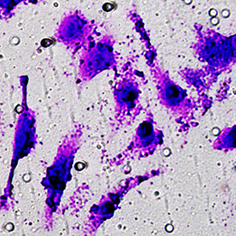

Supplement: Data S1 [file peerj-12-16935-s006.zip › The raw data of experiments/TRANSWELL/MDA-MB-468/invasion/si NC 3.jpg]

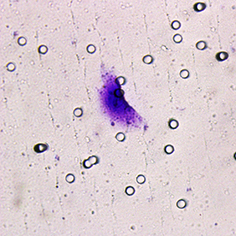

Supplement: Data S1 [file peerj-12-16935-s006.zip › The raw data of experiments/TRANSWELL/MDA-MB-468/migration/si ACP5 1.jpg]

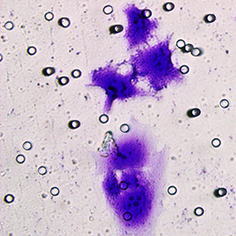

Supplement: Data S1 [file peerj-12-16935-s006.zip › The raw data of experiments/TRANSWELL/MDA-MB-468/migration/si ACP5 2.jpg]

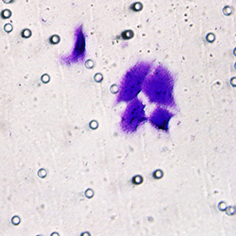

Supplement: Data S1 [file peerj-12-16935-s006.zip › The raw data of experiments/TRANSWELL/MDA-MB-468/migration/si ACP5 3.jpg]

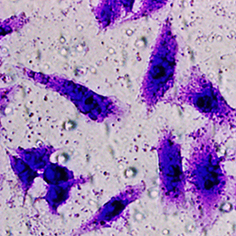

Supplement: Data S1 [file peerj-12-16935-s006.zip › The raw data of experiments/TRANSWELL/MDA-MB-468/migration/si NC 1.jpg]

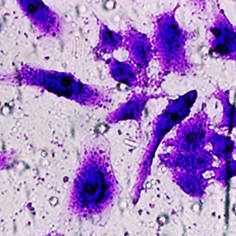

Supplement: Data S1 [file peerj-12-16935-s006.zip › The raw data of experiments/TRANSWELL/MDA-MB-468/migration/si NC 2.jpg]

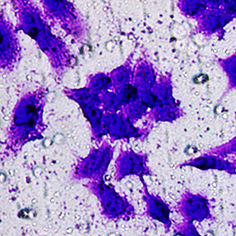

Supplement: Data S1 [file peerj-12-16935-s006.zip › The raw data of experiments/TRANSWELL/MDA-MB-468/migration/si NC 3.jpg]
