# Supplementary material for: Research and experimental verification on the mechanisms of cellular senescence in triple-negative breast cancer
Source: PeerJ. 2024 Feb 29;12:e16935. doi: 10.7717/peerj.16935 (PMC10909353; doi:10.7717/peerj.16935)
Supplement: Figure S2 [file peerj-12-16935-s002.pdf]

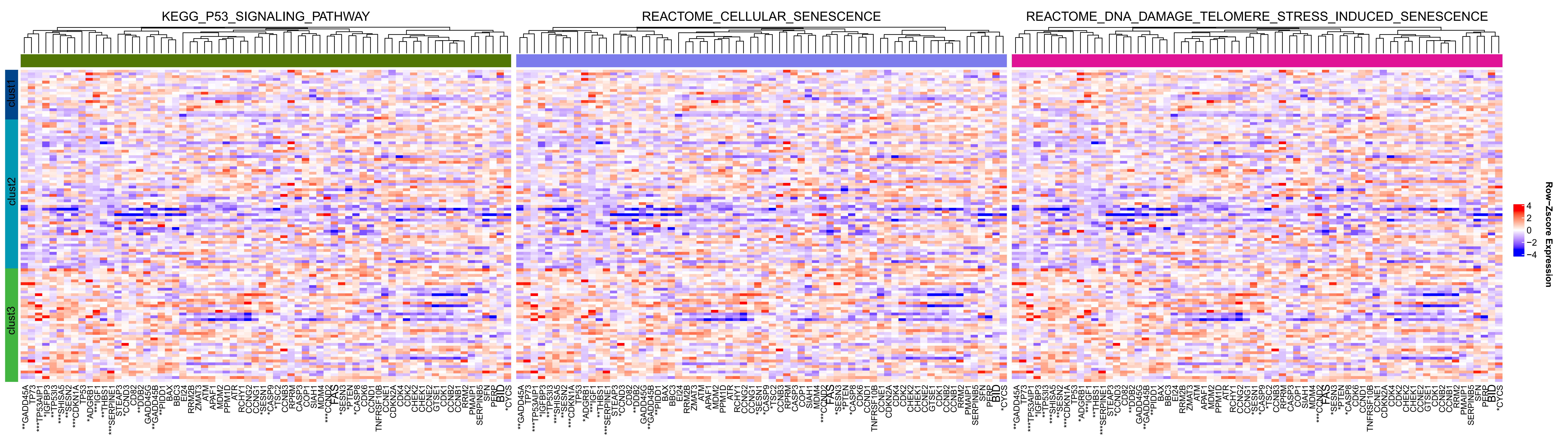
KEGG_P53_SIGNALING_PATHWAY
REACTOME_CELLULAR_SENESCENCE
REACTOME_DNA_DAMAGE_TELOMERE_STRESS_INDUCED_SENESCENCE
clust1
clust2
clust3
Row−Zscore Expression
4
2
0
−2
−4
