# Supplementary material for: Research and experimental verification on the mechanisms of cellular senescence in triple-negative breast cancer
Source: PeerJ. 2024 Feb 29;12:e16935. doi: 10.7717/peerj.16935 (PMC10909353; doi:10.7717/peerj.16935)
Supplement: Data S1 [file peerj-12-16935-s006.zip › The raw data of experiments/PCR results of signatures/PCR.pdf]

MMP28

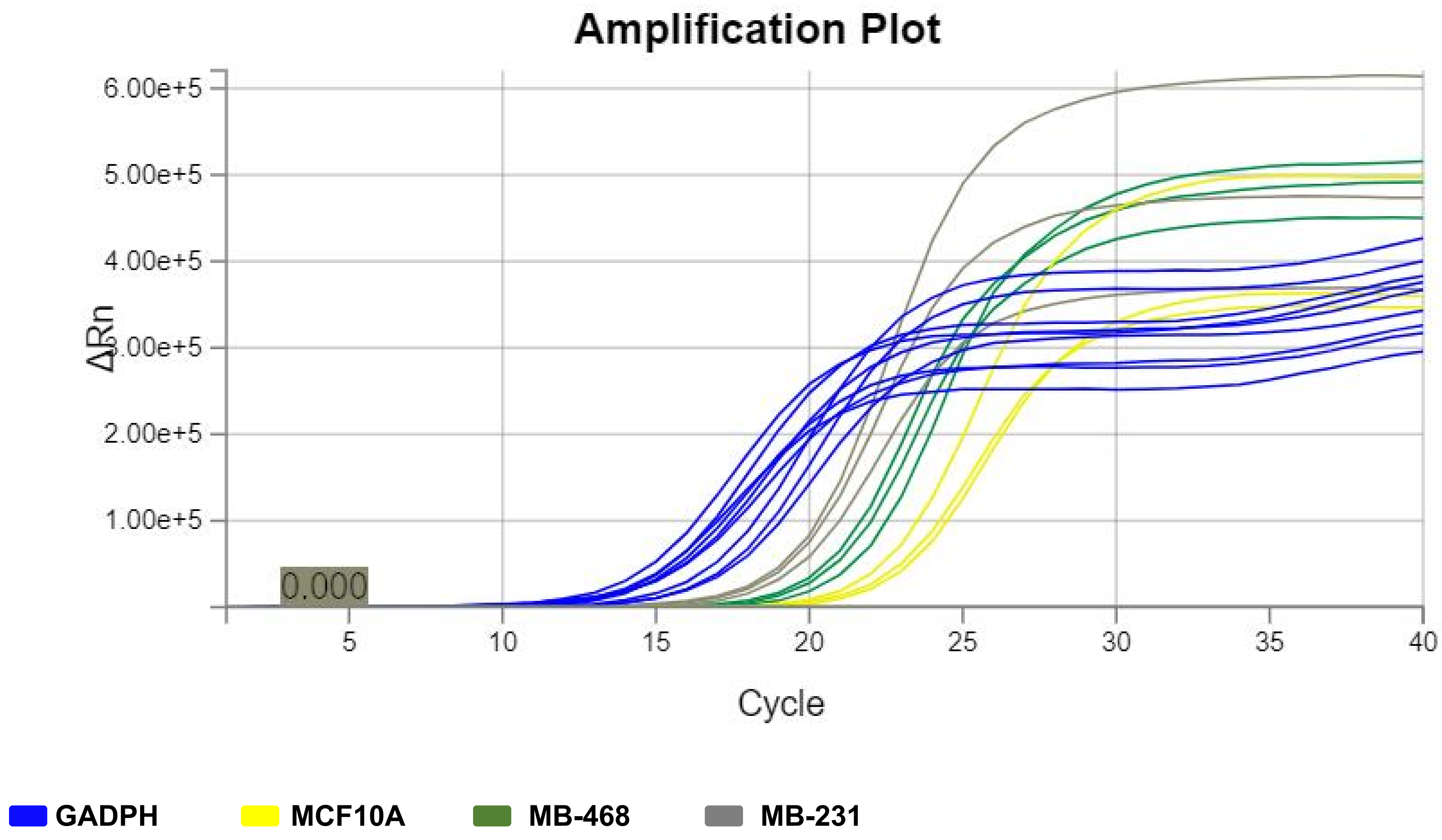

Amplification Plot
6.00e+5
5.00e+5
4.00e+5
3.00e+5
2.00e+5
1.00e+5
0.000
ΔRn
5
10
15
20
25
30
35
40
Cycle
GADPH
MCF10A
MB-468
MB-231

MMP28

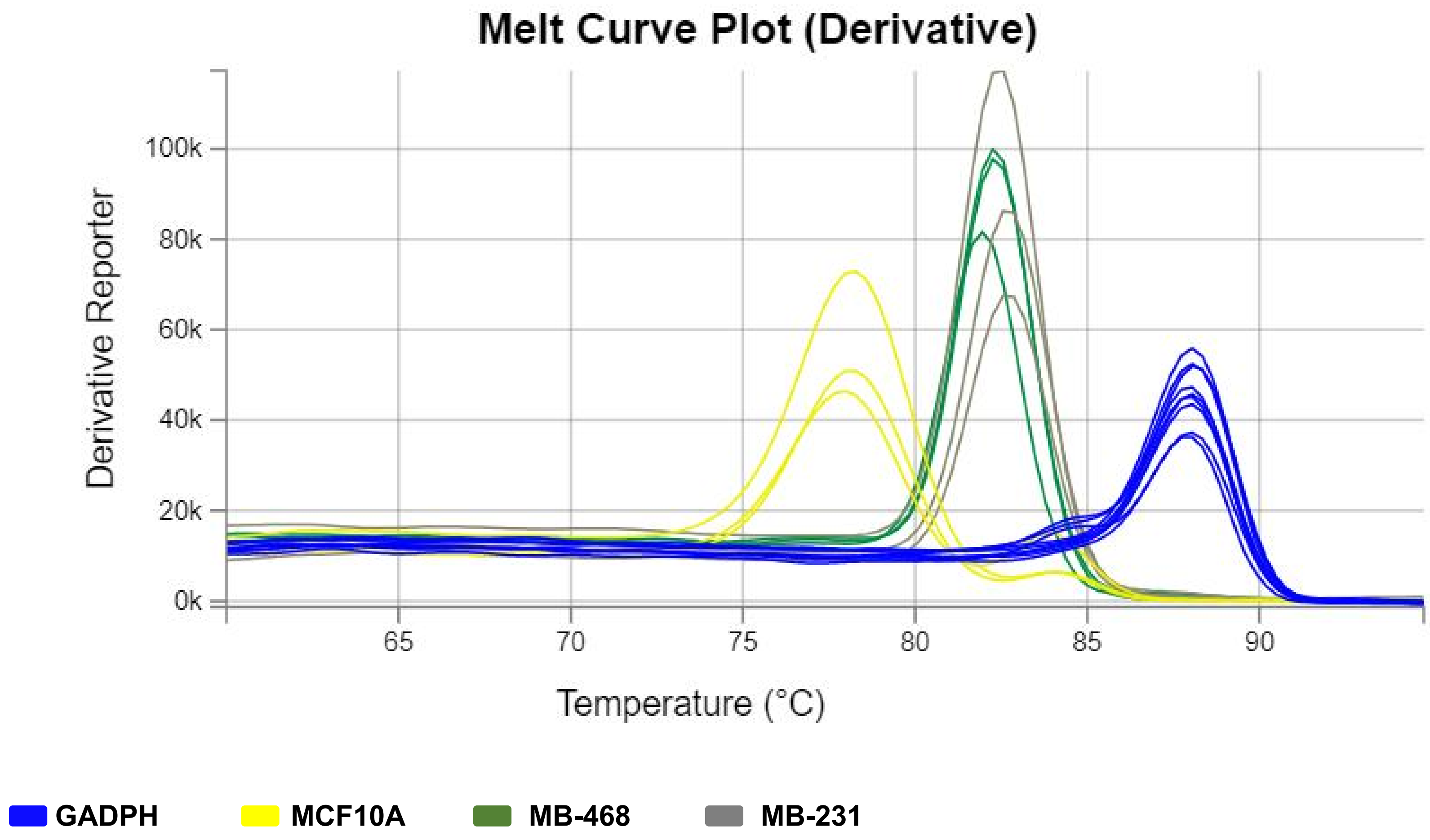

Melt Curve Plot (Derivative)
Derivative Reporter
100k
80k
60k
40k
20k
0k
65
70
75
80
85
90
Temperature (°C)
GADPH
MCF10A
MB-468
MB-231

CT83

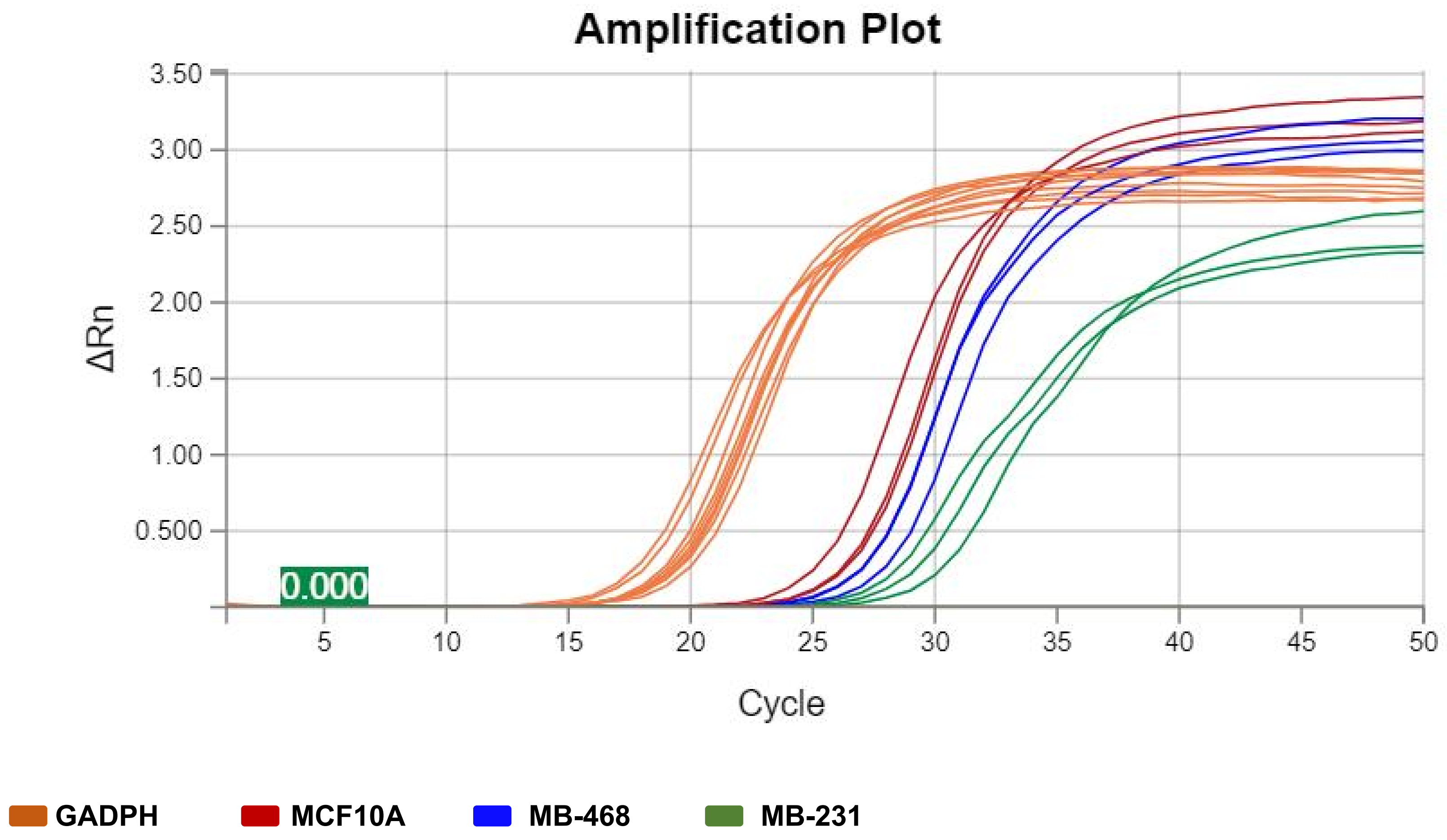
Amplification Plot
3.50
3.00
2.50
2.00
1.50
1.00
0.500
0.000
ΔRn
5
10
15
20
25
30
35
40
45
50
Cycle
GADPH
MCF10A
MB-468
MB-231

CT83

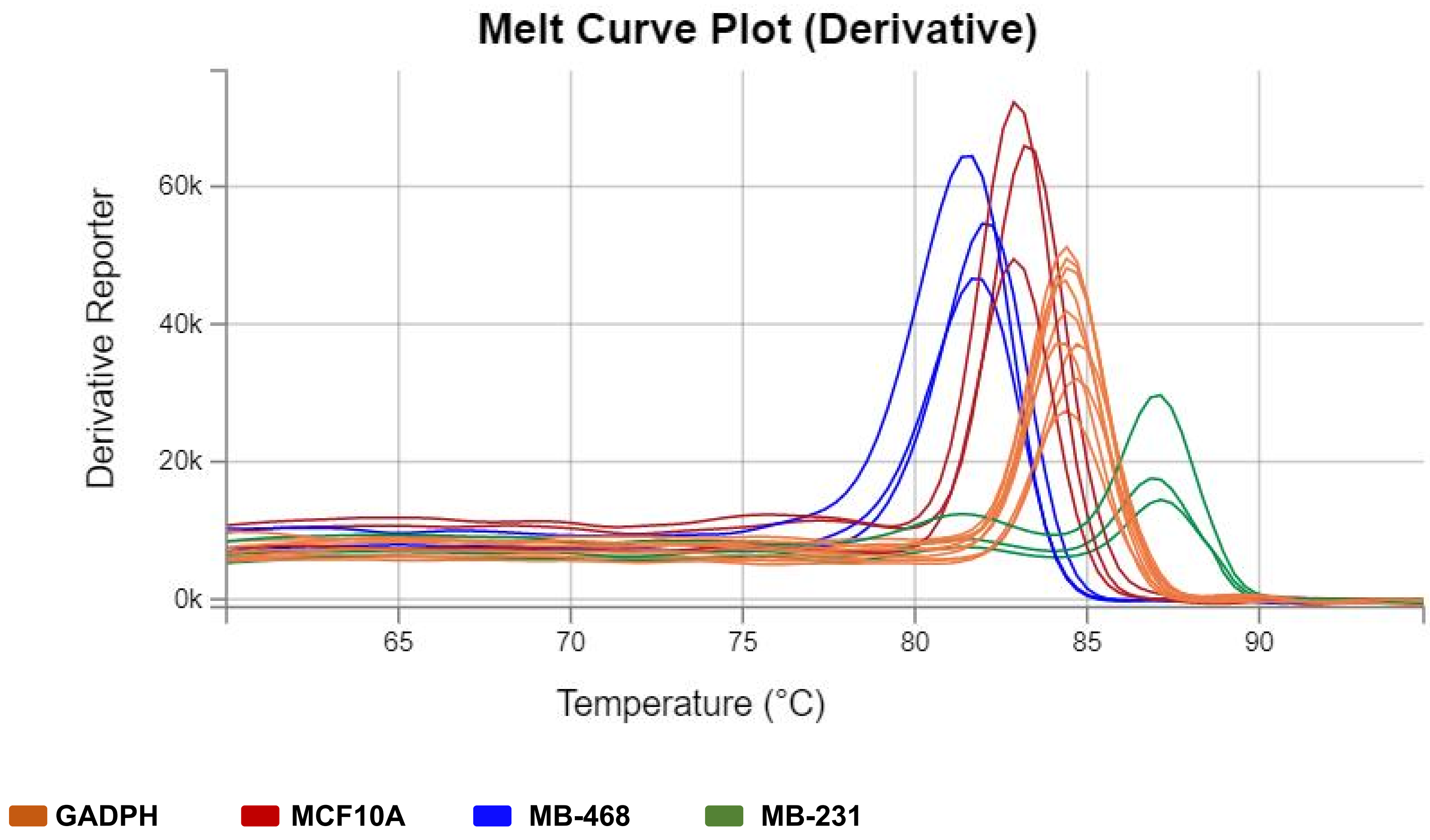

Melt Curve Plot (Derivative)
Derivative Reporter
60k
40k
20k
0k
65
70
75
80
85
90
Temperature (°C)
GADPH
MCF10A
MB-468
MB-231

ACP5

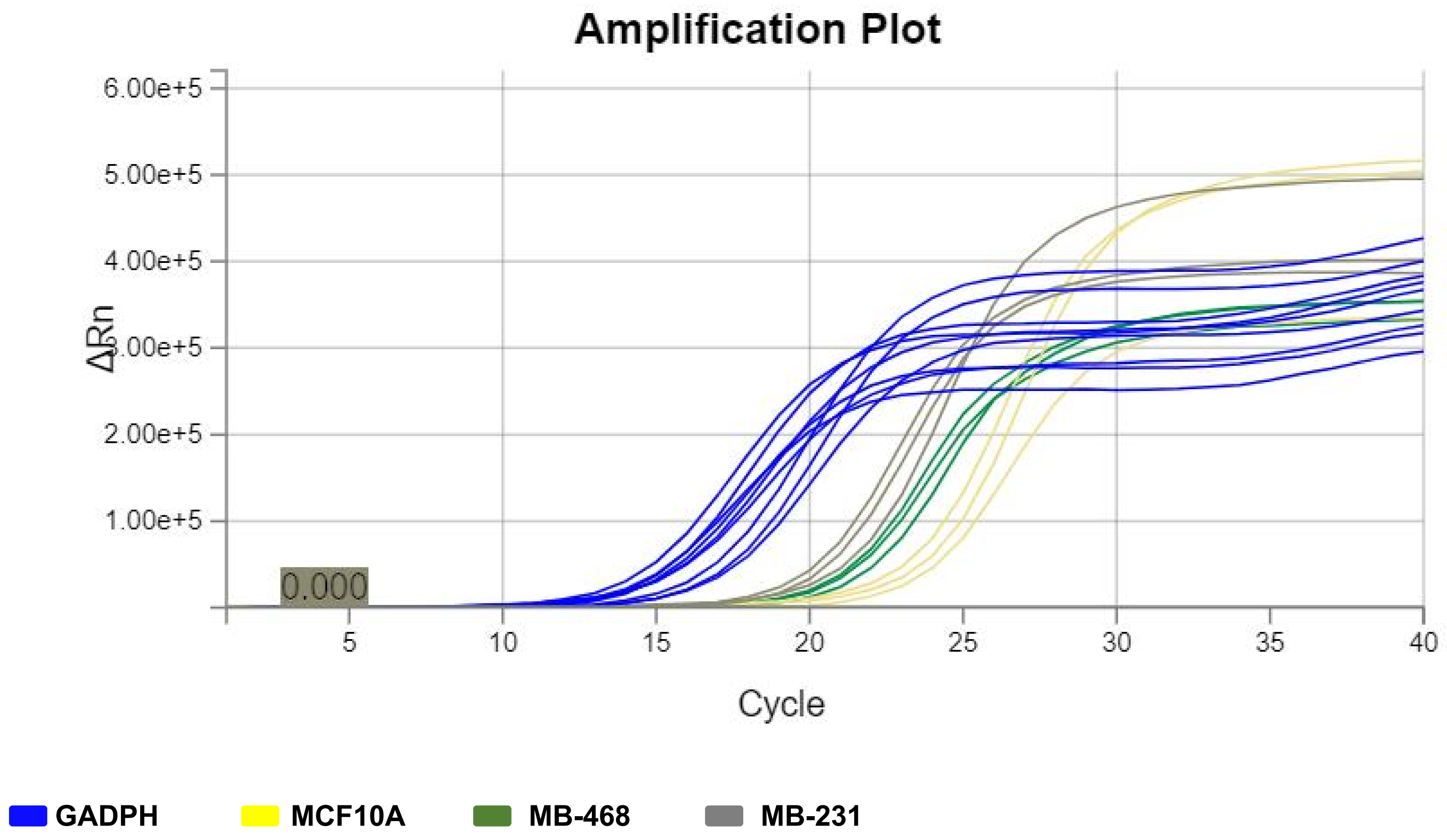
Amplification Plot
6.00e+5
5.00e+5
4.00e+5
3.00e+5
2.00e+5
1.00e+5
0.000
ΔRn
5
10
15
20
25
30
35
40
Cycle
GADPH
MCF10A
MB-468
MB-231

ACP5

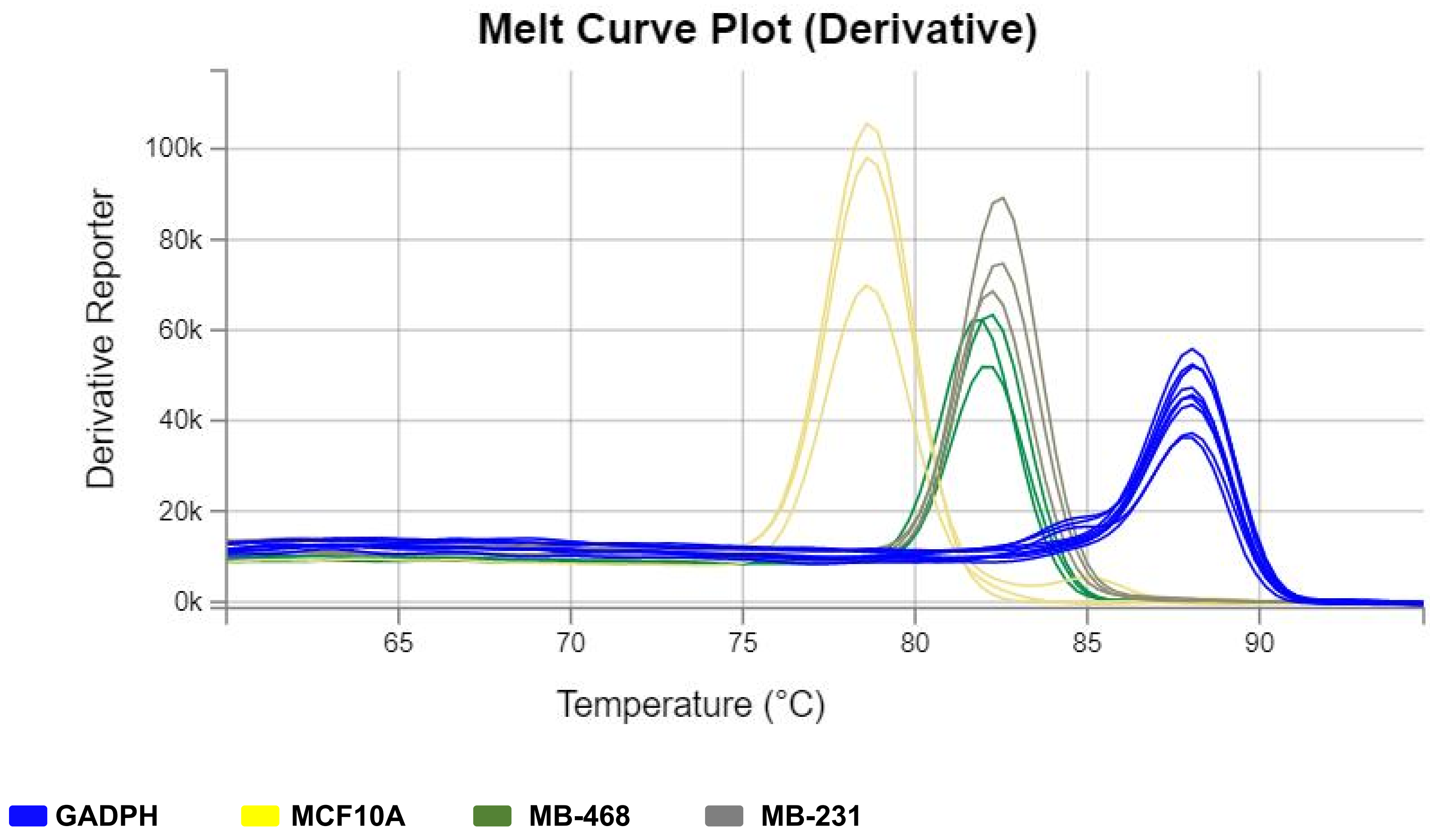

Melt Curve Plot (Derivative)
Derivative Reporter
100k
80k
60k
40k
20k
0k
65
70
75
80
85
90
Temperature (°C)
GADPH
MCF10A
MB-468
MB-231

KRT6A

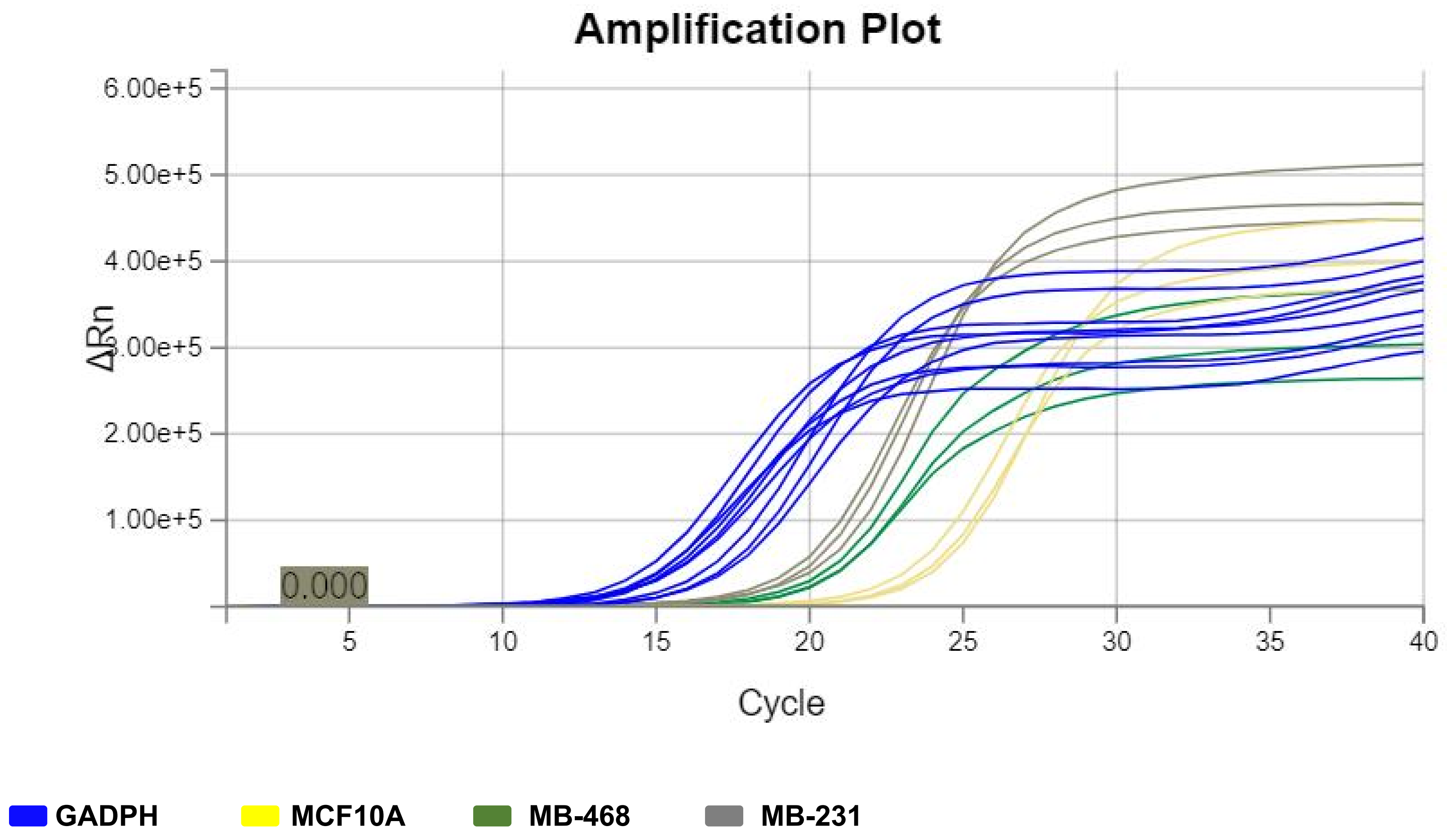
Amplification Plot
6.00e+5
5.00e+5
4.00e+5
3.00e+5
2.00e+5
1.00e+5
0.000
ΔRn
5
10
15
20
25
30
35
40
Cycle
GADPH
MCF10A
MB-468
MB-231

KRT6A

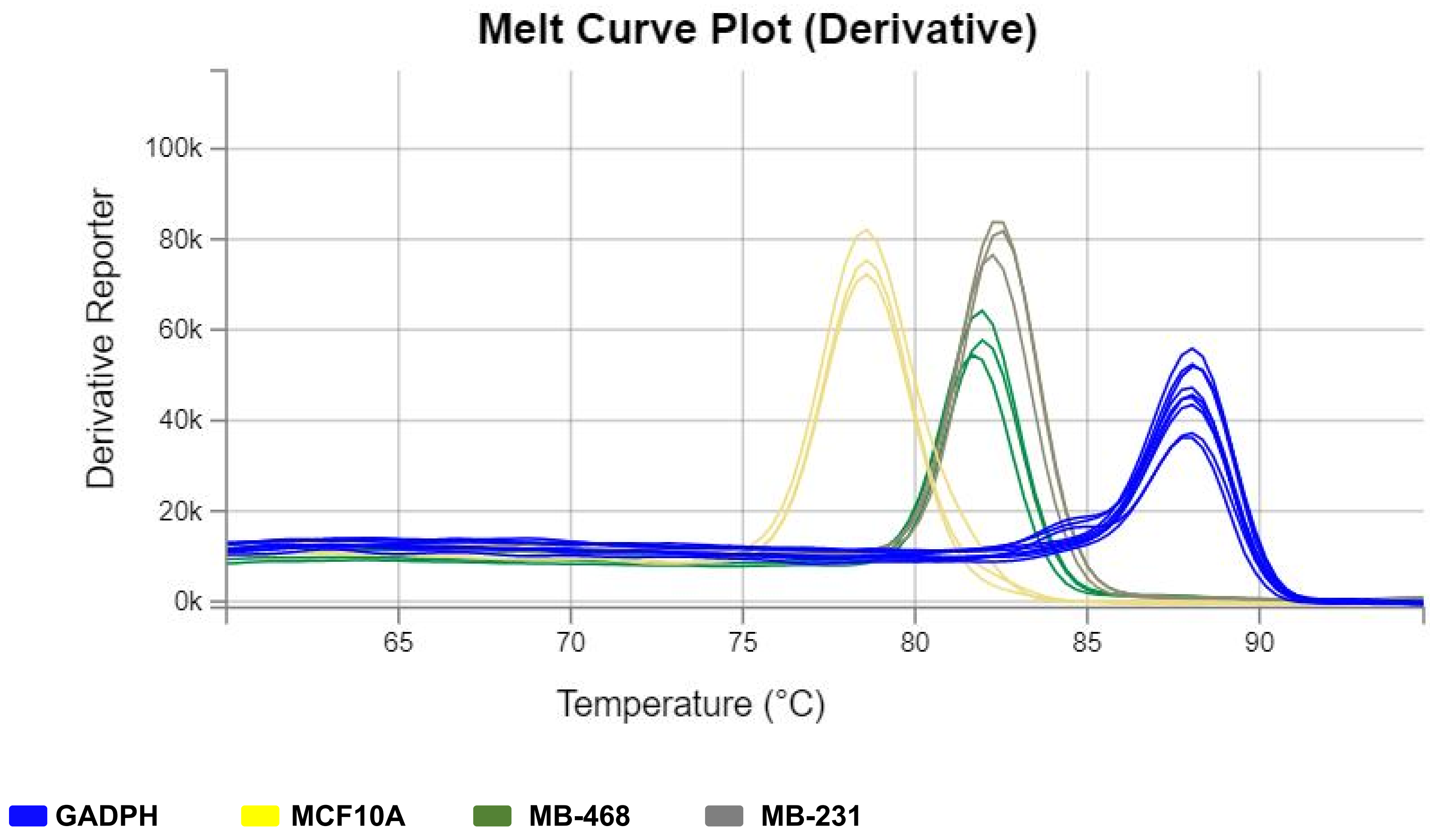
Melt Curve Plot (Derivative)
Derivative Reporter
100k
80k
60k
40k
20k
0k
65
70
75
80
85
90
Temperature (°C)
GADPH
MCF10A
MB-468
MB-231
